# In silico labeling reveals the time-dependent label half-life and transit-time in dynamical systems

**DOI:** 10.1186/1752-0509-6-13

**Published:** 2012-02-27

**Authors:** Thomas Maiwald, Julie Blumberg, Andreas Raue, Stefan Hengl, Marcel Schilling, Sherwin KB Sy, Verena Becker, Ursula Klingmüller, Jens Timmer

**Affiliations:** 1 Center for Systems Biology, Freiburg, Germany; 2 Department of Systems Biology, Harvard Medical School, Boston, USA; 3 Epilepsy Center, University Hospital Freiburg, Germany; 4 Department of Neuroscience, Children's Hospital, Harvard Medical School, Boston, USA; 5 Division Systems Biology of Signal Transduction, DKFZ-ZMBH Alliance, German Cancer Research Center, Heidelberg, Germany; 6 College of Pharmacy, University of Florida, Gainesville, USA; 7 Bioquant, Heidelberg University, Germany; 8 Freiburg Institute for Advanced Studies, University of Freiburg, Germany; 9 BIOSS Centre for Biological Signalling Studies, University of Freiburg, Germany; 10 Department of Clinical and Experimental Medicine, Linköping University, Sweden

## Abstract

**Background:**

Mathematical models of dynamical systems facilitate the computation of characteristic properties that are not accessible experimentally. In cell biology, two main properties of interest are (1) the time-period a protein is accessible to other molecules in a certain state - its half-life - and (2) the time it spends when passing through a subsystem - its transit-time. We discuss two approaches to quantify the half-life, present the novel method of *in silico labeling*, and introduce the *label half-life *and *label transit-time*. The developed method has been motivated by laboratory tracer experiments. To investigate the kinetic properties and behavior of a substance of interest, we computationally *label *this species in order to track it throughout its life cycle. The corresponding mathematical model is extended by an additional set of reactions for the labeled species, avoiding any double-counting within closed circuits, correcting for the influences of upstream fluxes, and taking into account combinatorial multiplicity for complexes or reactions with several reactants or products. A profile likelihood approach is used to estimate confidence intervals on the label half-life and transit-time.

**Results:**

Application to the JAK-STAT signaling pathway in Epo-stimulated BaF3-EpoR cells enabled the calculation of the time-dependent label half-life and transit-time of STAT species. The results were robust against parameter uncertainties.

**Conclusions:**

Our approach renders possible the estimation of species and label half-lives and transit-times. It is applicable to large non-linear systems and an implementation is provided within the PottersWheel modeling framework (http://www.potterswheel.de).

## Background

### Motivation

An increasing number of biological phenomena are described by mathematical models, specifically on the basis of biochemical reaction networks [1,2]. The dynamic properties of these networks are given by their model structure, kinetic parameters, initial values of the involved species, and externally specified input functions. The interpretation of an isolated element of the network, e.g. a certain rate constant, has only a limited meaning, because its effect can only be understood when taking the whole network context into account. We therefore seek to introduce two dynamical characteristics which have a physiological meaning, are intuitive to understand, and capture the system kinetics on a higher level of abstraction. The first characteristic, the *label half-life*, applies the half-life concept not to a species, but to a virtual label attached to the species. The second one, the *label transit-time*, is the time-period it takes for a fraction of labeled entities to pass through a subsystem of the network. Both quantities are calculated using a novel approach called *in silico labeling*, which is also introduced in the present work.

### In Silico Labeling and Species vs. Label Half-Life

In a laboratory tracer experiment, a substance is marked to better understand the kinetic properties of the dynamical system [3]. Different tracer substances have been used, e.g. radioactive iodine-125 [4,5] or green fluorescent protein-tagged proteins in combination with fluorescence recovery after photobleaching (FRAP) [6]. A good tracer does not hamper the flux of the substance, therefore one can assume that the flux of the tracer within a certain reaction is proportional to the flux of the original species. This is the key property of the *in silico labeling *approach, where an additional set of reactions is added to an existing mathematical model describing the kinetic behavior of a tracer, called the *label*. In contrast to real tracer experiments, the *in silico *method offers the opportunity to define dead-ends, avoid double-counting of cycling label, and to restrict the label to a sub-network of reactions. This allows asking specific questions about the original system, like how long it takes for 50% of the molecules of a substance to travel along a certain path, while in reality an alternative path may exist. In addition, predominant paths can be identified in deterministic models as has been done previously for stochastic systems [7].

**Figure 1:**
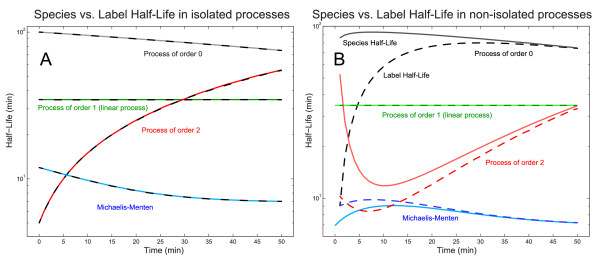
**Species vs. label half-life**. Panel A: The species half-life of a substrate *S *in the reaction *S *→ *P *is plotted for different reaction types (solid lines). Except for processes of order 1, the half-life is time-dependent. Since the substrate is not produced in further reactions, the label half-life (dashes) equals the species half-life. Panel B: The substrate *S *participates in a production (*A *→ *S*) and a processing (*S *→ *P *) reaction. Now, the species half-life differs except for a linear processing from the label half-life, because the label flux is proportional to the total flux of each reaction and is therefore affected by concentration changes through influx of *S*. Both panels: The species half-life has been determined analytically and numerically according to the methods section. Matlab scripts to reproduce the plots are available in the additional file 1.

Mathematically, the *half-life T*_1/2 _of a species is defined as the time-period until it reaches half of its initial amount assuming no influx. For clarity, we denote this time-period as the *species half-life (SHL)*. In non-isolated and non-linear processes, this time-period differs from the amount of time required for 50% of initially existing molecules to be processed. For this, we introduce the *label half-life (LHL)*, defined as the half-life of the label of a species. Equalities and differences between the species and label half-life are displayed in Figure 1 and proven in the methods section.

While for simple systems the species half-life can be determined analytically, the symbolic integration of a Michaelis-Menten kinetics leads to advanced mathematical calculations including the Lambert W function [8]. We therefore also provide an automatic and generally applicable numerical method to determine the species half-life.

### Label Transit-Time

Transit-times are discussed in a variety of fields and they are, for example, used to quantify how quickly food moves through the gastrointestinal tract [9]. When describing the dynamics of Markovian particles, the mean transit-time denotes the time spent on average in a subsystem [10], while the mean sojourn-time also takes into account the probability that the subsystem is entered at all [11]. In pharmacokinetics, the so-called mean residence time values [12] are estimated based on empirical data assuming linear kinetics [13]. Apart from linearity, no influx for the species of interest is permitted. Eventually, the estimation is only applicable to observable species. The computation of the mean residence time is accomplished by the ratio of the area under the first moment curve (AUMC) to the area under the curve (AUC) of the concentration-time profile of a drug [14].

We here introduce the *label transit-time *(LTT) from a source to a target pool in a chemical reaction network as the time-period after which 50% of all entities residing in the source pool at *t *= 0 have reached the target pool *at least once*. The exact path from source to target pool is not important in the unconditioned case. The LTT information could be valuable to estimate the time for a drug or an enzyme to reach its site of action.

### Extended Reaction Network

To determine the label half-life, it is important to distinguish entities residing in the source pool at *t *= 0 from other entities entering the source pool at later time-points. When calculating transit-times, this discrimination has to be applied to all pools and fluxes between source and target. To achieve this aim, the species of interest is computationally *labeled *and subsequently tracked throughout the dynamical model. The labeling is realized by an additional set of reactions describing the kinetic behavior of the labeled species, depending on the kind of time characteristic LHL or LTT, the source species, and potentially a target species.

In case of label half-life calculations, it is sufficient to create labeled reactions for all reactions in which the source species is a reactant. In fact, labeled reactions are prohibited if the source species is a product; this is to avoid double-counting the labeled species. In the case of transit-time calculations, for all original reactions in which labeled species are involved, a new labeled reaction is added. In all labeled reactions with the target species being the product, the label is removed and accumulated in an artificial pool which is used to determine when 50% of the existing label has reached the target.

The label stays virtually attached to a species throughout all modifications of the species, such as phosphorylation or relocalizations, e.g. shuttling into the nucleus. While the suggested approach can be implemented in a straightforward way for monomeric reaction networks with only up to one labeled reactant and product, for the general case where the reactions involve multiple reactants and products or where labeled species may form a polymer, a systematic book-keeping of all possible combinations of labeled and unlabeled species is required.

As motivated by laboratory tracer experiments the fluxes of the additional system are based on the corresponding fluxes in the original one, which is explained in detail in the methods section.

### Profile Likelihood-based Confidence Intervals

Recently, we suggested a profile likelihood-based approach to determine the confidence intervals on calibrated parameter values in mechanistic mathematical models [15]. The same reasoning can be applied in order to estimate confidence intervals for the time-dependent label half-life and transit-time characteristics.

### Implementation

All concepts have been implemented within the PottersWheel modeling and parameter estimation framework that is available from http://www.potterswheel.de[16] and have recently been applied by the authors to the mathematical models of the erythropoietin and epidermal growth factor receptors [17,18]. The application of the method within the PottersWheel framework is described in additional file 1.

In the next section, the proposed labeling method is illustrated for the JAK-STAT signal transduction pathway and afterwards described in detail. After proving the equality of species and label half-life for isolated or linear processes, a fitted model of the JAK-STAT pathway is used to determine the label half-life of unphosphorylated STAT and its label transit-time when cycling through the nucleus of a cell.

## Methods

### Illustration of the method

**Figure 2:**
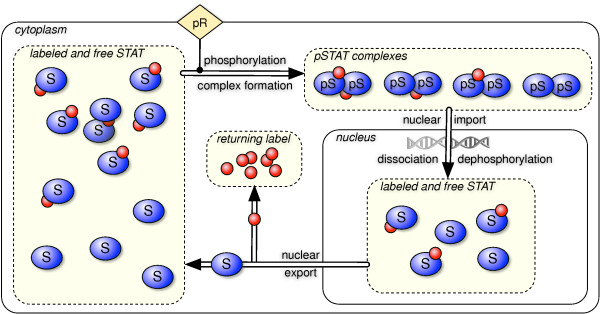
**In silico labeled JAK-STAT signaling pathway**. STAT molecules S (blue) are phosphorylated by an active receptor-kinase complex (pR) and form dimers (pS_-_pS). These dimers enter the nucleus, dissociate, and are subsequently dephosphorylated. Finally, the single STAT molecules re-enter the cytoplasm, where they can again be phosphorylated and thus continue the nuclear-cytoplasmic shuttling. The labeling approach is visualized by red spheres attached to the STAT molecules. At *t *= 0, all cytoplasmic STAT molecules are labeled. After the nuclear export, the label is removed from the molecule and enters the artificial pool of returned label. Consequently, an increasing fraction of cytoplasmic STAT molecules are not labeled which has to be considered in the calculation of fluxes for free and labeled entities. To determine the time-dependent label half-life and transit-time values, the labeling procedure is repeated for a series of time-points.

Figure 2 illustrates the *in silico *labeling approach for the JAK-STAT signal transduction pathway, where STAT molecules cycle between cytoplasm and nucleus. First, cytoplasmic STAT molecules (*S*) are phosphorylated (*pS*) by an active receptor (*pR*) and form dimers (*pS*_*pS*). The complexes enter the nucleus (*npS*_*npS*) where they act as transcription factors, disassociate and are dephosphorylated (*nS*) again. Finally, they return to the cytoplasm (*S*) and can be activated again. In order to determine the label half-life of cytoplasmic STAT and the label transit-time for a whole cycle, we set source and target species to unphosphorylated cytoplasmic STAT. At *t *= 0, all molecules of the source pool are labeled, symbolized by the small red spheres. The label is not removed until the target pool is reached, in this case when a STAT molecule leaves the nucleus. Then, the label is accumulated in an artificial pool of returned label and an unlabeled STAT molecule enters the cytoplasm. Over time, the fraction of labeled to free, unlabeled STAT molecules, *S^L^/S^F^*, decreases in the cytoplasm. The total flux $v_{1}^{T}$ of the first reaction, that is the phosphorylation of STAT molecules, can be divided into the flux $v_{1}^{L}$ of labeled and the flux $v_{1}^{F}$ of free STAT molecules. The fraction of the fluxes is set to match the fraction of labeled to free STAT molecules, by the following relationship:

(1) $$\frac{v_{1}^{L}}{v_{1}^{F}}:=\frac{S^{L}}{S^{F}}\text{with}\;v_{1}^{L}+v_{1}^{F}=v_{1}^{T}.$$

The label half-life of STAT at time-point *t *is given by

(2) $$\text{LH}{\text{L}}_{S}\left(t\right)=\arg_{t^{\prime }}\left(S^{L}\left(t^{\prime }\right)\overset{!}{=}\frac{S^{L}\left(t\right)}{2}\right)-t.$$

The label transit-time from STAT to STAT at time-point *t *can be derived from the time-profile of the returned label RL:

(3) $$\text{LT}{\text{T}}_{S\to S}\left(t\right)=\arg_{t^{\prime }}\left(\text{RL}\left(t^{\prime }\right)\overset{!}{=}\frac{S^{L}\left(t\right)}{2}\right)-t.$$

This procedure is repeated for a series of time-points *t *in order to determine LHL(*t*) and LTT(*t*) for all time-point of interest.

### Terminology

In the following we assume that the biological system is mathematically described by a set of reactions *r_j_*, 1 ≤ *j *≤ *n*, corresponding to a set of coupled differential equations. The concentration change of each entity *x_i_*, 1 ≤ *i ≤ m*, is the sum over all fluxes of reactions where it appears as a product minus the sum over all fluxes of reactions where it appears as a reactant, mathematically [19]

(4) $${ẋ}_{i}=\overset{n}{\underset{j\neq i}{\sum }}a_{ij}v_{j}-\overset{n}{\underset{j\neq i}{\sum }}b_{ij}v_{j}\text{ for }i\leq 1\leq m.$$

Here, *v_j _*describes the flux of reaction *j, a_ij _*≥ 0 the stoichiometry of *x_i _*as a product in reaction *j *and *b_ij _*≥ 0 the stoichiometry of *x_i _*as a reactant in reaction *j*. We use the same symbol for an entity and its concentration, [*x_i_*] ≐ *x_i_*. The time-profile of each species can then be calculated for given initial values $x_{i}^{0}=x_{i}\left(t_{0}\right)$ and potentially driving input functions *u_k_*(*t*). The flux *v_j _*of reaction *j *may be a non-linear function of one or more species concentrations *x_i _*and externally defined *u_k_*. To improve readability, we omit explicitly denoting the time-dependency, i.e. *x_i_*(*t*) is rather written as *x_i_*.

### Analytical and numerical half-life calculation

The half-life of a species *x_i _*of interest is determined by extending the differential equation network (4) by one equation for an artificial quantity *y *depending only on the outfluxes of *x_i_*,

(5) $$ẏ=-\overset{n}{\underset{j\neq i}{\sum }}b_{ij}v_{j}$$

with initial value $y^{0}=x_{i}^{0}.$ The whole system (4, 5) is solved either analytically or numerically and the species half-life of *x_i _*is given by *T*_1/2 _for which

(6) $$y\left(T_{1/2}\right)=\frac{y^{0}}{2}.$$

Note that a half-life characterizes the decay of a quantity, independent of any production rates. Therefore, all influx contributions are neglected in equation (5). In general, only linear processes possess a constant half-life. Otherwise, the half-life depends on the initial concentration $x_{i}^{0}$ and is therefore time-dependent. In this case, the above procedure is repeated for a series of different initial time-points *t*_0_. In a numerical integration, it is important to limit the maximum integrator step size for an accurate approximation of the *y*^0^/2 threshold crossing.

The half-life of a species *x_i _*is only partially related to the time it takes for 50% of an experimental tracer to leave the source pool. The two values coincide if *x_i _*has either no influx or when the outflux from *x_i _*is described by a linear process, which will be proved in the next two subsections. Therefore, we suggest the *in silico labeling half-life *as a means to determine a time-characteristic which is motivated by laboratory tracer experiment with the additional property to avoid tracer-double counting in kinetic cycles.

### In silico labeling half-life for isolated processes

For simplicity, we assume that the species of interest *x *∈ {*x*_1_, . . . , *x_m_*} is consumed only in one reaction. In *in silico *labeling, the flux of the corresponding label *z *depends on the outflux of *x *by

(7) $$ż=-\frac{z}{x_{i}}v_{out}.$$

The *in silico *labeling half-life of *x *is defined as the time when *z *drops to *z*_0_*/*2. We will show that this time equals the species half-life of *x *if its influx *v_in _*is zero. This property is independent from the amount of initially labeled entities, i.e. it holds for any *z*_0_*/x*_0 _∈ ℝ^+^:

Proof:

Let *x *be determined by the processing with an unknown, potentially non-linear outflux *v_out _*and no influx *v_in _*= 0, i.e. *v *= *v_out_*,

$$ẋ=-v\text{ and }x_{0}=x\left(0\right).$$

Then, the kinetics of the label species *z*(*t*) is given by

(8) $$ż=-\frac{z}{x}v\left(x\right)\text{ and }z_{0}=z\left(0\right).$$

It can be shown that the factor $\frac{z}{x}$ is constant:

$$\begin{array}{ccc} \frac{d}{dt}\left(\frac{z}{x}\right) & =\frac{żx-zẋ}{x^{2}} & \\ & =\frac{-\frac{z}{x}vx+zv}{x^{2}}\\ & =0. \end{array}$$

Since this relation holds also true for *t *= 0, the proportionality constant is given by $f=\frac{z_{0}}{x_{0}}.$ Then, equation (8) reads

$$ż\left(t\right)=-fv\text{ and }z_{0}=fx_{0}.$$

Both processes *x*(*t*) and *z*(*t*) share the same half-life *T*_1/2_, since

$$x\left(T_{1/2}\right)=\frac{x_{0}}{2}\Rightarrow z\left(T_{1/2}\right)=fx\left(T_{1/2}\right)=f\frac{x_{0}}{2}=\frac{z_{0}}{2}\text{ , q}\text{.e}\text{.d}\text{.}$$

This relation does not hold for processes with *v_in _*≠ 0, because the fraction *z/x *becomes time-dependent as the labeling gets diluted, except for linear outfluxes as shown in the next section.

### In silico labeling for linear processes

In this section, we prove that the label half-life coincides with the half-life of a species *x *which is produced by an unknown, potentially non-linear influx *v_in _*and is consumed by a linear process.

Proof:

Let  be given by an unknown, potentially non-linear influx *v_in _*and a linear outflux, *kx*,

$$ẋ=v_{in}-kx$$

Then, the analytical half-life of *x *can be determined via

$$\begin{array}{ccc} ẏ & = & -ky\\ \Rightarrow y\left(t\right) & = & y_{0}e^{-kt}\\ \Rightarrow T_{1/2}\left(x\right) & = & \frac{\text{ln}\left(2\right)}{k} \end{array}$$

For the labeled system *z *it holds that

$$\begin{array}{c} \begin{array}{ccc} ż & = & \frac{z}{x}v_{out}\\ & = & -\frac{z}{x}kx\\ & = & -kz\\ \Rightarrow z\left(t\right) & = & z_{0}e^{-kt}\\ \Rightarrow T_{1/2}\left(z\right) & = & \frac{\text{l}n\left(2\right)}{k}\text{ , q}\text{.e}\text{.d} \end{array} \end{array}$$

### Creating the Extended Reaction Network

Some entities *x_i _*belong to the group of tracked, i.e. potentially labeled entities. Let us assume that they are given by *x*_1_, . . . , *x_α _*and untracked ones by *x*_*α+*1_, . . . , *x_m_*. Further, it can be assumed without loss of generality that (1) *x*_1_, . . . , *x*_*γ *≤ α _are not complexes consisting of two or more tracked single entities, and (2) that the tracked single entities within each complex *x*_*γ*+1_, . . . , *x_α _*belong to the set *x*_1_, . . . , *x_γ_*. In the JAK-STAT example, *S, pR_S*, and *pS *belong to *x*_1_, . . . , *x_α _*and *pS*_*pS *to *x*_*α*+1_,. . . , *x_m _*as it contains two labeled single entities *pS*.

### Creating additional entities x*^LF^*

A new set of labeled or free entities **x***^LF ^*is created based on the original **x**, by applying the following rules:

• Start with an empty set, **x***^LF ^*= {}

• Single entities: For each *x_i _*∈{*x*_1_, . . . , *x_γ_*}, **x***^LF ^*is enlarged by a labeled $x_{i}^{L}$ and a free $x_{i}^{F}$ version of the original entity

• Complex entities: Each complex *x_i _*∈ {*x*_*γ*+1_, . . . , *x*_α_} is decomposed into $n_{1}^{i}x_{1},\ldots ,n_{\gamma }^{i}x_{\gamma }.$ Due to the combinatorial multiplicity,

(9) $$2^{\sum_{j=1}^{\gamma }n_{j}^{i}}$$

possible combinations using labeled $x_{j}^{L}$ and free $x_{j}^{F}$ entities are created, taking into account the order of the elements in the original complex *x_i_*, and are added to **x***^LF^*. The complex *pS*___*pS *for instance leads to the four new complexes *pS^F^_pS^F^, pS^L^_pS^F^, pS^F^_pS^L^*, and *pS^L^_pS^L^*.

### Creating additional reactions r*^LF^*

In order to create a new set of reactions **r***^LF ^*, the combinatorial multiplicity has to be applied not only to complexes but also to the ordered lists of reactants and products. Suppose an ordered list *I *of entities from the set {*x_i_*}_1 ≤ *i*≤*α *_with possible repetition, as for example the reactants of the reaction *A *+ *A *+ *pA*_*pA *→ *A_A_pA_pA *corresponding to *I *= (*A, A, pA_pA*). Summing up all single reactants and elements of the complexes leads to p single entities, in this case *p *= 4. Taking into account all combinations of labeled and free entities, 2*^p ^*different lists can be derived, in the example

$$I_{1}=\left(A^{F},\;A^{F},\;pA^{F}\text{\_}pA^{F}\right),\;I_{2}=\left(A^{L},\;A^{F},\;pA^{F}\text{\_}pA^{F}\right),\;...,\;\text{and}\;I_{16}=\left(A^{L},\;A^{L},pA^{L}\text{\_}pA^{L}\right).$$

Without loss of generality, only the first *δ *reactions of the original system are assumed to affect a tracked entity. In these reactions, at least one reactant or product is a tracked entity. Then, a new set of reactions **r***^LF ^*can be established. Starting with the empty set **r***^LF ^*= {}, for each reaction *r_i _*∈ **{***r*_1_, . . . , *r_δ_***} **with one or more reactants of tracked entities,

1. all reactants and products not belonging to the group of tracked entities are removed,

2. the combinatorial multiplicity approach is applied to the ordered list *I *of the remaining reactants leading to $I_{1},...,I_{2^{p}}$,

3. 2*^p ^*reactions are added to **r***^LF ^*with reactants $I_{1},...,I_{2^{p}}$ and the corresponding products.

4. the fluxes $v_{1}^{LF},\ldots ,v_{j}^{LF}$ of the new reactions are given by

(10) $$v_{j}^{LF}=\frac{\prod_{x_{k}^{LF}\in I_{j}}x_{k}^{LF}}{\prod_{x_{k}\in I}x_{k}}\;v_{i},\;\;1\leq j\leq 2^{p}.$$

Note that again the same symbol has been used for the entity name and its concentration. The sum over all weighting factors is 1.

Reactions *r_i _*∈ {*r*_1_, . . . , *r_δ_*}without reactants produce only free entities, which simplifies the conversion of *r_i _*before adding to **r***^LF^*: All untracked entities are removed, all *x_i _*are replaced by $x_{i}^{F}$, and the flux is again given by equation (10).

When calculating the label half-life, products that coincide with the initially labeled entity are replaced by the corresponding free entity. This corresponds to removing the label and is necessary to avoid double-counting and to exclude upstream fluxes.

In order to calculate the label transit-time, entities entering the target pool must be released from their labeling, again, to avoid double-counting. Therefore, all labeled target entities are replaced in the reaction network **r***^LF ^*by their free counterparts. At the same time, a new product is added to those reactions where the target entity is a product to accumulate the returned label, RL.

### Calculating the Label Half-Life and Transit-Time

Since the label half-life and transit-time characteristics are time-dependent, the label is not only *injected *at time-point 0, but the procedure is repeated for a series of time-points *t *(let *x_i _*be the source species):

1. Set all initial values for labeled entities and RL, if available, to 0. Set the initial value of free entities to the value of their counterpart in the original network.

2. Numerically integrate the ordinary differential equations corresponding to the extended reaction network {**r**, **r***^LF^*} from 0 to *t*.

3. Apply a complete labeling of the source species: Set $x_{i}^{L}\left(t\right)=x_{i}\left(t\right)$ and $x_{i}^{F}\left(t\right)=0.$ This step corresponds to the label *injection*.

4. Continue the numerical integration.

Threshold crossing at *t*" of the time-profiles $x_{i}^{L}\left(t^{\prime }>t\right)$ and RL(*t' > t*) with $x_{i}^{L}\left(t\right)/2$ defines the label half-life and label transit-time as *t*"-*t*, respectively. The threshold crossing is determined by linear interpolation of the discrete samples given by the numerical integration.

### Profile Likelihood-based Confidence Intervals

We recently suggested a profile likelihood-based approach to determine simultaneous and separate confidence intervals for calibrated unknown model parameters [15]. In order to determine confidence intervals for the calculated label half-life and transit-times, the above procedure is not only repeated for a series of time-points, but also for a series of parameter settings. Each setting corresponds to one extreme point on the multi-dimensional manifold of acceptable parameter values, where one parameter has reached a lower or upper confidence threshold. By plotting all LHL or LTT profiles into one axis and creating an envelope between the largest and lowest values, a confidence interval for LHL and LTT is given.

### Analytic half-lives for simple, isolated processes

The half-life *T*_1*/*2_(*t*) of simple and isolated biochemical reactions can be calculated analytically. Except for first-order processes, it usually depends on the concentration *x*_0 _= *x*(*t*_0_) at the time-point of interest *t*_0 _and is therefore time-dependent:

(11) $$\text{Process of order 0: }T_{1/2}=\;\frac{x_{0}}{2k}$$

(12) $$\text{Process of order 1: }T_{1/2}=\;\frac{ln\left(2\right)}{2k}$$

(13) $$\text{Process of order }2:\;T_{1/2}=\;\frac{1}{x_{0}\cdot k}$$

(14) $$\text{Process of order n}>1:\;T_{1/2}=\;\frac{2^{n-1}-1}{\left(n-1\right)k\cdot x_{0}^{n-1}}$$

(15) $$\text{Michaelis - Menten}:\;T_{1/2}=\;\frac{\ln\left(2\right)K_{m}+x_{0}/2}{V_{max}}$$

The half-life calculation for a process of order *n >*1 with $ẋ=-kx^{n}$ is based on the integral form

(16) $$\frac{1}{x^{n-1}}=\frac{1}{x_{0}^{n-1}}+\left(n-1\right)kt.$$

In order to calculate the half-life for Michaelis-Menten kinetics, $ẋ=-V_{max}x/\left(K_{m}+x\right),$ the following integral form is used which has been derived in [20], for *x*(*t*) with known *x*_0 _at *t *= *t*_0_:

(17) $$x_{0}-x+K_{m}\ln\left(\frac{x_{0}}{x}\right)=V_{max}\left(t-t_{0}\right)$$

Panel A of Figure 1 displays the analytic results and their numerical approximation.

## Results

In this section, the *in silico *labeling approach is applied to the JAK-STAT signaling pathway. The following mass action-based mechanistic model of the pathway has been calibrated to immunoblot measurements for Epo-stimulated BaF3-EpoR cells (model motivated by and data taken from [21]):

(18) $$d\left(\text{S}\right)/dt\;=\;-k_{1}\;\text{S}\;\text{pR}+k_{5}\;\text{nS}$$

(19) $$d\left(\text{pS}\right)/dt\;=\;k_{1}\;\text{S}\;\text{pR}-2\;k_{2}\;\text{pS}\;\text{pS}$$

(20) $$d\left(\text{pS\_pS}\right)/dt\;=\;k_{2}\;\text{pSpS}-k_{3}\text{pS}\text{\_}\text{pS}$$

(21) $$d\left(\text{npS\_npS}\right)/dt\;=\;k_{3}\text{pS\_pS}-k_{4}\text{npS\_npS}$$

(22) $$d\left(\text{nS}\right)/dt\;=\;2\;k_{4}\;\text{npS\_npS}-k_{5}\;\text{nS}$$

A smoothing spline approximation of the phosphorylated receptor served as the input function *pR*(*t*) triggering the phosphorylation of STAT (*S *→ *pS*). After dimerization (*pS *+ *pS *→ *pS_-_pS*), the complexes enter the nucleus (*pS*_*pS *→ *npS_npS*). Then they dissociate and are dephosphorylated (*npS_npS *→ *nS *+ *nS*). Finally, single STAT molecules leave the nucleus again (*nS *→ *S*). Model parameters were estimated using a Levenberg-Marquardt approach and the PottersWheel modeling software. The pools of total and phosphorylated cytoplasmic STAT have been used as observation functions. The kinetic parameters were estimated as *k*_1 _= 1.37, *k*_2 _= 0.22, *k*_3 _= 0.63, *k*_4 _= 0.59, and *k*_5 _= 0.59. The initial value of *S *was calibrated to 0.96 and the scaling factors for the observables to 1.45 for *pS_-_obs *and 0.98 for *S_-_obs*.

### Labeled system

**Figure 3:**
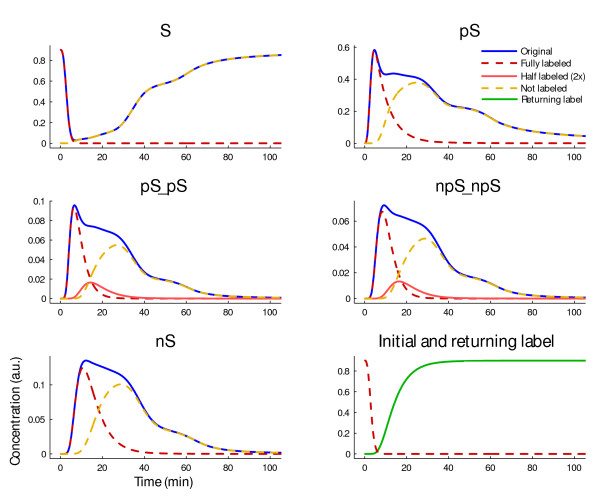
**Time-courses of the original and labeled system**. At *t *= 0, all STAT molecules are labeled and the original system (solid blue) coincides with the labeled one (dashed red). After the initial signal wave, STAT molecules return from the nucleus to the cytoplasm. Since the label transit-time for a complete cycle of cytoplasmic STAT is investigated, the returning molecules release their label, which is counted in an artificial entity (bottom right, green). Unlabeled molecules are depicted in yellow. Solid rose lines depict half labeled species, i.e. dimers of a labeled and an unlabeled molecule. They are shown only once, since the trajectory for example of *pS^L^*_-_*pS^F ^*is identical to the one of *pS^F^*_-_*pS^L^*.

**Figure 4:**
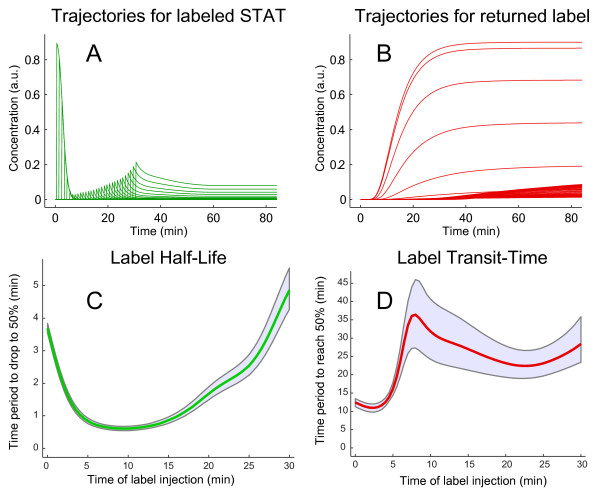
**Time-dependent label half-life and transit-time of cytoplasmic unphosphorylated STAT**. A: In order to determine the label half-life and transit-time, a family of labeled STAT trajectories is calculated. For trajectory *i*, the amount of *S*(*t_i_*) label is injected into the system as *S^L ^*at *t_i_*, with 0 ≤ *t_i _*≤ 30. Therefore, the upper limit of subplot A matches the time-course of S for *t ≤ *30 in Fig 3. B: Shown are the corresponding trajectories for returned label. The label half-life for trajectory *i *is the time period until $S_{i}^{L}\left(t\right)$ drops below $S_{i}^{L}\left(t_{i}\right)/2.$ C: The shortest label half-life is given as 0.6 ± 0.1 minutes. D: The label transit-time for a complete cycle of STAT molecules through the nucleus is the time-period until the returning label exceeds half of its initial value. Here, the minimum was estimated as 12 ± 2 minutes. Previously, it has been shown that the sojourn time of a single STAT5 molecule in the nucleus is about 6 minutes [21]. Our results indicate that on (median) average, a STAT5 molecule spends equal times in each compartment and requires about 12 min for one cycle from the cytoplasm to the nucleus and back. The 95% confidence intervals have been determined using the profile likelihood approach (PLE) and are displayed as grey envelope curves.

In order to determine the label half-life and transit-time of STAT, *S *is both, the initially labeled entity and the target pool. The flux of the label is illustrated in Figure 2. The time-courses of the original (solid blue) and labeled system (dashed red) are compared in Figure 3. In the beginning, both systems behave in the same manner. Then, the first wave of STAT molecules return from their cycle through the nucleus. Since they loose their label, the amount of labeled cytoplasmic STAT does not recover in contrast to the amount of STAT. After ~ 13 minutes, 50% of the initially labeled STAT molecules passed the nucleus at least once, as shown by the artificial pool of the returned label. The bimodal behavior of pSTAT exemplifies the first original signal wave and the secondary cycling effects. The *in silico *labeling approach allowed for discrimination between these two dynamics. In order to determine the transit-time for *t >*0, the label is injected at a series of time-points, which is visualized in Figure 4.

### Label half-life and transit-time

Figure 4 depicts the label half-life of STAT as calculated from the time-course of *S^L^*. It reaches a minimum of 0.6 ± 0.1 minutes after ten minutes compared to a half-life of approximately 3 to 4 minutes at the starting point of the time-course analysis. For later time-points, the stimulus decreases (not shown) and STAT is no longer phosphorylated, resulting in an increased label half-life of STAT molecules. The minimum label transit-time for a complete cycle of STAT molecules was estimated as 12 ± 2 minutes.

### Profile likelihood-based confidence intervals

In order to investigate how uncertainties in calibrated model parameter values propagate to the estimated time-characteristics, we applied the profile likelihood approach on an identifiable version of the model. The kinetic parameters involved in phosphorylation (*k*_1_), dimerization (*k*_2_), nuclear import (*k*_3_) and export (*k*_5_) were systematically varied consecutively within four orders of magnitude between 0.01*k_fit _*and 100*k_fit_*, with *k_fit _*being the parameter value for the best fit. For each variation, the other free parameters were calibrated resulting in a profile likelihood estimation (see Fig. S2 in additional file 1). All parameter settings corresponding to a crossing of the profile likelihood with the *X*^2^-threshold of the separate 95% confidence interval are used to recalculate the label half-life and transit-time. Figure 4C and 4D display the LHL and LTT 95% confidence interval by envelope curves. In case of the label half-life of cytoplasmic STAT, the confidence interval is very narrow allowing the LHL estimation within ± 0.1 minute for a range of label injection times between *t *= 0 and *t *= 20 minutes. The label transit-time has a wider confidence interval reflecting the larger number of reactions involved in a complete cycle of shuttling STAT.

## Discussion and Conclusions

In this paper, the half-life of a species has been compared conceptually, analytically, and numerically to the half-life of a label in a hypothetical tracer experiment. Two time-characteristics, the label half-life and label transit time have been introduced, which capture the kinetics of a dynamical system on a higher level than e.g. single rate constants. Calculation of the time-characteristics and their profile likelihood-based confidence intervals for an identifiable pathway model showed that the approach is robust against parameter uncertainties. The quantities are calculated based on the novel *in silico *labeling method, which relies on an extended reaction network taking into account constraints concerning double-counting, upstream fluxes and combinatorial multiplicity. Our model-based *in silico *approach allows for insights into reaction networks that cannot be determined experimentally.

The proposed method provides important information for a wide spectrum of biological applications ranging from cell biology and pharmacokinetics to population dynamics. We applied it to a non-linear model of the cellular JAK-STAT signaling pathway, which allowed for calculating the time-dependent label half-life and transit-time of cytoplasmic STAT.

In summary our approach enables to calculate the amount of time a molecule spends in a certain state or compartment and therefore provides novel insights into the temporal scale of networks. This knowledge will have profound impact on drug design, as it offers the possibility to predict the life-time of a specific molecule and provides a basis to improve drug targeting.

## Authors' contributions

TM and JB: Definition of the biological question, initiation, development, and implementation of the proposed method, writing of the manuscript. AR: Development of the proposed method, writing of the manuscript. SH, MS, SS, VB, UK, JT: Definition of the biological question, initiation of the method, critical discussion and contribution to manuscript. All authors read and approved the final manuscript.

## Supplementary Material

Additional file 1 **Application within PottersWheel**. This additional file contains MATLAB scripts to run various tasks related to the in silico labeling approach. http://www.biomedcentral.com/imedia/4654854926777309/supp1.pdf.. Click here for file
