# Supplementary material for: In silico labeling reveals the time-dependent label half-life and transit-time in dynamical systems
Source: BMC Syst Biol. 2012 Feb 27;6:13. doi: 10.1186/1752-0509-6-13 (PMC3395849; doi:10.1186/1752-0509-6-13)
Supplement: Additional file 1 — Application within PottersWheel. This additional file contains MATLAB scripts to run various tasks related to the in silico labeling approach. http://www.biomedcentral.com/imedia/4654854926777309/supp1.pdf.. [file 1752-0509-6-13-S1.PDF]

Application within PottersWheel
Additional file for:
In silico labeling reveals the time-dependent label half-life and transit-time in dynamical systems

Thomas Maiwald*[1,2], Julie Blumberg*[3,4] , Andreas Raue[1] , Stefan Hengl[1] , Marcel Schilling[5] , Sherwin K. B. Sy[6] , Verena Becker[2,5,7] , Ursula Klingmüller[5,7] and Jens Timmer[1,8,9,a]

[1]Center for Systems Biology, Freiburg, Germany
[2]Department of Systems Biology, Harvard Medical School, Boston, USA
[3]Epilepsy Center, University Hospital Freiburg, Germany
[4]Department of Neuroscience, Children's Hospital, Harvard Medical School, Boston, USA
[5]Division Systems Biology of Signal Transduction, DKFZ-ZMBH Alliance, German Cancer Research Center, Heidelberg, Germany
[6]College of Pharmacy, University of Florida, Gainesville, USA
[7]Bioquant, Heidelberg University, Germany
[8]Freiburg Institute for Advanced Studies, University of Freiburg, Germany
[9]BIOSS Centre for Biological Signalling Studies, University of Freiburg, Germany
[a]Department of Clinical and Experimental Medicine, Linköping University, Sweden

Email: Thomas Maiwald*- thomas_maiwald@hms.harvard.edu;

*Corresponding author

*These authors contributed equally.

Abstract

This supplemental information contains MATLAB scripts to run various tasks related to the in silico labeling approach. It requires installation of the PottersWheel toolbox version 2.2 or above. The used toolbox functions begin either with the prefix "pw", "General_", or "Tools_". In the first case, the function belongs to the PottersWheel application programming interface (API) and is documented on www.potterswheel.de/Pages/API. Many PottersWheel functions possess a corresponding button or menu entry in the graphical user interfaces.

1 Script to generate Fig. 1 of the main text

1.1 Isolated processes

```
models = {'MichaelisMenten', 'OrderZeroModel', 'OrderOneModel', 'OrderTwoModel'};
analyticalHL = {};
labelHL = {};
```

```
for i=1:length(models)

pwClear
config = pwGetConfig;
config.plotting.showX = true;
config.plotting.showY = false;
config.plotting.showZ = false;
config.integration.integrator = 14;
config.integration.useJacobian = true;
pwSetConfig(config);

m = pwGetEmptyModel();
m.ID = models{i};
m = pwAddX(m, 'bS', 20);
m = pwAddX(m, 'cS', 0);

switch models{i}
    case 'MichaelisMenten'
        m = pwAddX(m, 'bS', 1);
        m = pwAddR(m, {'bS'}, {'cS'}, { }, 'C', [] , 'k1*r1/(k2+r1)', {'Vmax','Km'});
        m = pwAddK(m, 'Vmax', 0.1);
        m = pwAddK(m, 'Km'  , 1  );

    case 'OrderZeroModel'
        m = pwAddR(m, {'bS'}, {'cS'}, {  }, 'C' , [] , 'k1', {'k'});
        m = pwAddK(m, 'k', 0.1);

    case 'OrderOneModel'
        m = pwAddR(m, {'bS'}, {'cS'}, {  }, 'C' , [] , 'k1*r1', {'k'});
        m = pwAddK(m, 'k', 0.02);

    case 'OrderTwoModel'
        m = pwAddR(m, {'bS'}, {'cS'}, {  }, 'C' , [] , 'k1*r1*r1', {'k'});
        m = pwAddK(m, 'k', 0.01);
end

pwAddModel(m);
pwSim;
pwCombine;
pwArrange;
pwDraw;
[matrix, IDs] = pwGetPlottingData({'tFine', 'xFine'});
t = matrix(:, Mex_FindPosition('time', IDs));
S = matrix(:, Mex_FindPosition('bS', IDs));

switch models{i}
    case 'MichaelisMenten'
        Km = pwGetParameterValuesByID('Km');
        Vmax = pwGetParameterValuesByID('Vmax');
        analyticalHL{i} = (log(2) * Km + S / 2)/Vmax;
```

```
    case 'OrderZeroModel'
        k = pwGetParameterValuesByID('k');
        analyticalHL{i} = S/(2*k);
    case 'OrderOneModel'
        k = pwGetParameterValuesByID('k');
        analyticalHL{i} = log(2)/(k) * ones(size(S));
    case 'OrderTwoModel'
        k = pwGetParameterValuesByID('k');
        analyticalHL{i} = 1./(S*k);
    otherwise
        error('Unknown model: %s', model);
end

pwSelect(1);
sourceIDs = 'bS';
targetIDs = 'cS';
labeledBasicIDs = 'S';
allowCycling = false;
values = [];
additionalDerivedVariables = {'bSLabel + bSFree', 'bSLabel_plus_bSFree', 'bS', 'bS'};
tInjection = 10;
pwInSilicoLabeling(sourceIDs, targetIDs, labeledBasicIDs, allowCycling, ...
  values, additionalDerivedVariables, tInjection);
pwSelect('last');
pwCombine;
pwArrange;
pwDraw

variationValues = 0:1:50;
[labelHL{i}, transitTimes] = pwHalfLife('bSLabel','cSLabel','Injection__bSLabel', ...
  variationValues, 0.5, 0.5);

end

%% Plot

figure(10)
subplot(1,1,1,'replace');
ind = find(t<=max(variationValues));
colors = General_GetColorCells(10, [], 2);
colors = {colors{[3 5 6 7]}};

for i=1:length(models)
    h = semilogy(t(ind), analyticalHL{i}(ind), '-', 'LineWidth', 2);
    set(h, 'Color', colors{i});
    hold on
end
legend(models, 0);
for i=1:length(models)
    semilogy(variationValues, labelHL{i}, 'k--', 'LineWidth', 2);
end
```

```
title(sprintf('Half-Life for %s', Tools_join(', ', models)), 'FontSize', 14);
xlabel('Time', 'FontSize', 14);
ylabel('Half-Life', 'FontSize', 14);
[xLim yLim] = General_GetLimitsOfPlotData;
xlim([xLim(1) - 0.05 * (xLim(2) - xLim(1)) xLim(2) + 0.05 * (xLim(2) - xLim(1))]);
ylim([yLim(1) - 0.01 * (yLim(2) - yLim(1)) yLim(2) + 0.10 * (yLim(2) - yLim(1))]);
```

1.2 Not isolated processes

```
models = {'MichaelisMenten', 'OrderZeroModel', 'OrderOneModel', 'OrderTwoModel'};
analyticalHL = {};
labelHL = {};

for i=1:length(models)

pwClear
config = pwGetConfig;
config.plotting.showX = true;
config.plotting.showY = false;
config.plotting.showZ = false;
config.integration.integrator = 14;
config.integration.useJacobian = true;
config.integration.opt.MaxStep = 0.1;
pwSetConfig(config);

m = pwGetEmptyModel();
m.ID = models{i};
m = pwAddX(m, 'aS', 20);
m = pwAddX(m, 'bS', 0);
m = pwAddX(m, 'cS', 0);

m = pwAddR(m, {'aS'}, {'bS'}, { }, 'C', [] , 'k1*r1',         {'A_to_S'});

switch models{i}
    case 'MichaelisMenten'
        m = pwAddX(m, 'aS', 1);
        m = pwAddR(m, {'bS'}, {'cS'}, { }, 'C', [] , 'k1*r1/(k2+r1)', {'Vmax','Km'});
        m = pwAddK(m, 'Vmax', 0.1);
        m = pwAddK(m, 'Km'  , 1  );

    case 'OrderZeroModel'
        m = pwAddR(m, {'bS'}, {'cS'}, { }, 'C' , [] , 'k1', {'k'});
        m = pwAddK(m, 'k', 0.1);

    case 'OrderOneModel'
        m = pwAddR(m, {'bS'}, {'cS'}, { }, 'C' , [] , 'k1*r1', {'k'});
        m = pwAddK(m, 'k', 0.02);
```

```
    case 'OrderTwoModel'
        m = pwAddR(m, {'bS'}, {'cS'}, { }, 'C' , [] , 'k1*r1*r1', {'k'});
        m = pwAddK(m, 'k', 0.01);
end

pwAddModel(m);
%pwAddModel('OrderZeroModel');
pwSim;
pwCombine;
%pwArrange;
pwDraw;
[matrix, IDs] = pwGetPlottingData({'tFine', 'xFine'});
t = matrix(:, Mex_FindPosition('time', IDs));
S = matrix(:, Mex_FindPosition('bS', IDs));

switch models{i}
    case 'MichaelisMenten'
        Km = pwGetParameterValuesByID('Km');
        Vmax = pwGetParameterValuesByID('Vmax');
        analyticalHL{i} = (log(2) * Km + S / 2)/Vmax;
    case 'OrderZeroModel'
        k = pwGetParameterValuesByID('k');
        analyticalHL{i} = S/(2*k);
    case 'OrderOneModel'
        k = pwGetParameterValuesByID('k');
        analyticalHL{i} = log(2)/(k) * ones(size(S));
    case 'OrderTwoModel'
        k = pwGetParameterValuesByID('k');
        analyticalHL{i} = 1./(S*k);
    otherwise
        error('Unknown model: %s', model);
end

pwSelect(1);
sourceIDs = 'bS';
targetIDs = 'cS';
labeledBasicIDs = 'S';
allowCycling = false;
values = [];
additionalDerivedVariables = {'bSLabel + bSFree', 'bSLabel_plus_bSFree', 'bS', 'bS'};
tInjection = 10;
pwInSilicoLabeling(sourceIDs, targetIDs, labeledBasicIDs, allowCycling, values, ...
   additionalDerivedVariables, tInjection);
pwSelect('last');
pwCombine;
pwArrange;
pwDraw

variationValues = 1:1:50;
[labelHL{i}, transitTimes] = pwHalfLife('bSLabel','cSLabel','Injection__bSLabel', ...
   variationValues, 0.5, 0.5);
```

```
end

%% Plot

figure(10)
subplot(1,1,1,'replace');
ind = find(t<=max(variationValues));
colors = General_GetColorCells(10, [], 2);
colors = {colors{[3 5 6 7]}};

legendEntries = {};
for i=1:length(models)
    h = semilogy(t(ind), analyticalHL{i}(ind), '-', 'LineWidth', 2);
    set(h, 'Color', colors{i});
    hold on
    legendEntries{i} = ['Species HL ', models{i}];
end
legendEntries{end+1} = 'Label HL';
for i=1:length(models)
    semilogy(variationValues, labelHL{i}, 'b-', 'LineWidth', 2);
    if i==1
        legend(legendEntries, 0);
    end
end

title(sprintf('Half-Life for %s', Tools_join(', ', models)), 'FontSize', 14);
xlabel('Time', 'FontSize', 14);
ylabel('Half-Life', 'FontSize', 14);
[xLim yLim] = General_GetLimitsOfPlotData;
set(gca, 'XLim', [xLim(1) - 0.05 * (xLim(2) - xLim(1)) xLim(2) + 0.05 * (xLim(2) - xLim(1))]);
ylim([6 100])
```

2 Generating Fig. 4 of the main text

2.1 Time-courses of the original and labeled JAK-STAT pathway

```
% Content of file PW_Macro_Labeling_JakStat.m
% It is possible to copy and paste the below commands into the Matlab command window.

pwClear;
config = pwGetConfig;
config.model.splitReversibleReactions = true;
config.integration.useFastIntegration = true;
config.integration.integrator = 1;
config.optimization.method = 2;
config.optimization.fitInLogParameterSpace = true;
config.plotting.showU = false;
config.plotting.showX = true;
config.plotting.showY = true;
```

```
config.plotting.showZ = false;
pwSetConfig(config);

m = pwGetEmptyModel();
m.ID = 'JakStat';
m = pwAddX(m, 'S', 1);
m = pwAddR(m, {'S'},       {'pS'},     {'pR'},'C', [], 'k1*r1*m1', {'S_to_pS'});
m = pwAddR(m, {'pS', 'pS'},{'pS_pS'},  {},    'C', [], 'k1*r1*r2', {'dimerization'});
m = pwAddR(m, {'pS_pS'},   {'npS_npS'},{},    'C', [], 'k1*r1',    {'nuc_import'});
m = pwAddR(m, {'npS_npS'}, {'nS','nS'},{},    'C', [], 'k1*r1',    {'dimer_break'});
m = pwAddR(m, {'nS'},      {'S'},      {},    'C', [], 'k1*r1',    {'nuc_export'});
m = pwAddY(m, 'pS + 2 * pS_pS', 'pS_obs');
m = pwAddY(m, 'S + pS + 2 * pS_pS', 'S_obs');
m = pwAddU(m, 'pR', 'steps', [-1 0], [0 1]);
pwAddModel(m);

pwAddDataFromLibrary('JAK-STAT5-Swameye-2003-Dataset-01.xls', '.')
pwCombine;
pwArrange;
pwFit;

pwInSilicoLabeling('S', 'S', 'S');
pwSelect('last');
pwAddDataFromLibrary('JAK-STAT5-Swameye-2003-Dataset-01.xls', '.')
pwCombine;
pwArrange;
pwDraw;
```

3 Profile Likelihood estimation

In order to determine confidence intervals on the label half-life and transit-time, the profile likelihood of the calibrated JAK-STAT model has to be estimated. This is done using the graphical user interfaces `pwPLEgui` (see Fig. 1) and `pwHalfLifeGUI`. The profile-likelihood is plotted for four parameters in Fig. 2.

3.1 Initialization

```
% PottersWheel Macro to apply the in silico labeling approach onto
% the JAK-STAT signal transduction pathway.

pwClear

%% Configuration

config = pwGetConfig;
config.data.uCalculation = 2;
config.data.yStdCalculation = 2;
config.model.splitReversibleReactions = true;
config.integration.useFastIntegration = false;
config.integration.integrator = 14;
config.integration.opt.RelTol = 1e-6;
```

```
config.integration.opt.AbsTol = 1e-8;
config.integration.useJacobian = true;
config.integration.calcJacobian = true;
config.optimization.useJacobian = true;
config.optimization.calcJacobian = true;
config.optimization.method = 2;
config.optimization.fitInLogParameterSpace = true;
config.optimization.trustregion.TolFun = 1e-8;
config.optimization.trustregion.TolX = 1e-8;
config.analyses.PLE.allowBetterOptimum = false;
config.analyses.PLE.thresholdMode = 2;
config.analyses.PLE.fitBeforePLE = true;
config.plotting.showU = false;
config.plotting.showX = true;
config.plotting.showY = true;
config.plotting.nFine = 1000;
config.plotting.showModelInfo = false;
config.plotting.showParInfo = false;
config.plotting.menuBar = true;
config.plotting.toolBar = true;
pwSetConfig(config);


%% Load model and data

m = pwGetEmptyModel();
m.ID = 'JakStat';
m = pwAddX(m, 'S', 1);
m = pwAddR(m, {'S'},       {'pS'},     {'pR'},'C', [], 'k1*r1*m1', {'S_to_pS'});
m = pwAddR(m, {'pS', 'pS'},{'pS_pS'},  {},    'C', [], 'k1*r1*r2', {'dimerization'});
m = pwAddR(m, {'pS_pS'},   {'npS_npS'},{},    'C', [], 'k1*r1',    {'nuc_import'});
m = pwAddR(m, {'npS_npS'}, {'nS','nS'},{},    'C', [], 'k1*r1',    {'dimer_break'});
m = pwAddR(m, {'nS'},      {'S'},      {},    'C', [], 'k1*r1',    {'nuc_export'});
m = pwAddY(m, 'pS + 2 * pS_pS', 'pS_obs');
m = pwAddY(m, 'S + pS + 2 * pS_pS', 'S_obs');
m = pwAddU(m, 'pR', 'steps', [-1 0], [0 1]);
pwAddModel(m);

matrix = [...
0 0 0.074 1 0.084 0.01713
2 0.3315 0.05 0.9275 0.046 0.145
4 0.8645 0.066 0.7923 0.038 0.2442
6 0.9635 0.07 0.7778 0.032 0.7659
8 0.9279 0.065 0.7053 0.033 1
10 0.8162 0.051 0.6522 0.037 0.8605
12 0.7553 0.053 0.5894 0.039 0.7829
14 0.768 0.051 0.5894 0.04 0.5705
16 0.8416 0.04 0.6377 0.03 0.6217
18 0.768 0.04 0.6425 0.028 0.331
20 0.801 0.048 0.6908 0.03 0.3388
25 0.7832 0.052 0.6908 0.031 0.3116
```

```
30 0.8086 0.054 0.7585 0.032 0.05062
40 0.4888 0.055 0.8068 0.04 0.02504
50 0.2782 0.044 0.9275 0.046 0.01163
60 0.2553 0.071 0.971 0.082 0];

colNames = {'Time', 'pS_obs', 'stdCol-pS_obs', 'S_obs', 'stdCol-S_obs', 'pR'};
ds = pwGetDataSet(matrix, colNames, 'JAK-STAT-Swameye-2003-Dataset-01-tMax250');

pwAddData(ds, true)

%% Single Fit

pwCombine;
pwDraw;
pwArrange;
pwFit;


%% Set fixed parameters and fit limits

pwSetFixedParameters({'S_C1', 'scale_pS_obs_C1', 'scale_S_obs_C1', 'dimer_break'});
pwSetFitLimitsAroundCurrentValues(100);


%% Start Profile Likelihood Estimation

pwPLEgui;
```

References

1. Swameye I, Müller TG, Timmer J, Sandra O, Klingmüller U: **Identification of nucleocytoplasmic cycling as a remote sensor in cellular signaling by databased modeling.** *Proc Natl Acad Sci U S A* 2003, **100**(3):1028–1033, [http://dx.doi.org/10.1073/pnas.0237333100].

2. Raue A, Kreutz C, Maiwald T, Bachmann J, Schilling M, Klingmüller U, Timmer J: **Structural and practical identifiability analysis of partially observed dynamical models by exploiting the profile likelihood.** *Bioinformatics* 2009, **25**(15):1923–1929, [http://dx.doi.org/10.1093/bioinformatics/btp358].

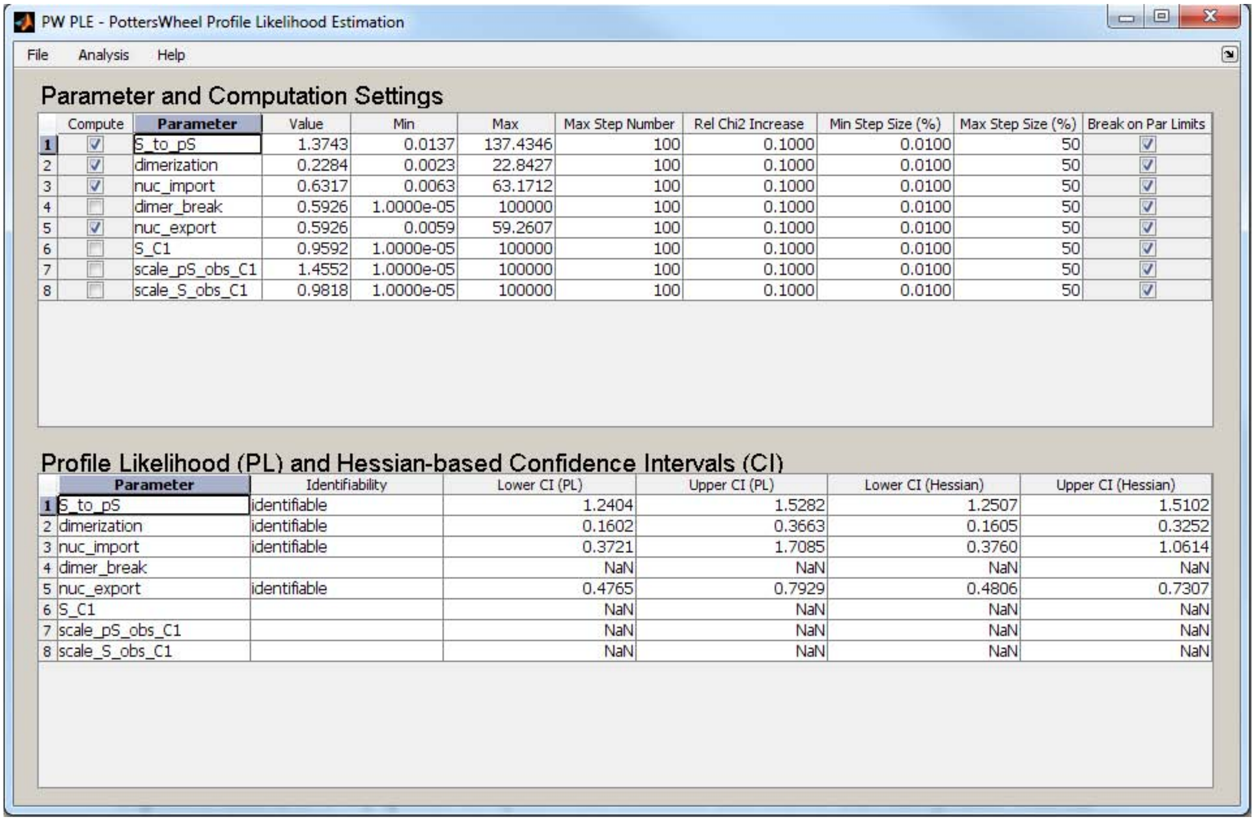

PW PLE - PottersWheel Profile Likelihood Estimation

File Analysis Help

Parameter and Computation Settings

	Compute	Parameter	Value	Min	Max	Max Step Number	Rel Chi2 Increase	Min Step Size (%)	Max Step Size (%)	Break on Par Limits
1	☑	S_to_pS	1.3743	0.0137	137.4346	100	0.1000	0.0100	50	☑
2	☑	dimerization	0.2284	0.0023	22.8427	100	0.1000	0.0100	50	☑
3	☑	nuc_import	0.6317	0.0063	63.1712	100	0.1000	0.0100	50	☑
4	☐	dimer_break	0.5926	1.0000e-05	100000	100	0.1000	0.0100	50	☑
5	☑	nuc_export	0.5926	0.0059	59.2607	100	0.1000	0.0100	50	☑
6	☐	S_C1	0.9592	1.0000e-05	100000	100	0.1000	0.0100	50	☑
7	☐	scale_pS_obs_C1	1.4552	1.0000e-05	100000	100	0.1000	0.0100	50	☑
8	☐	scale_S_obs_C1	0.9818	1.0000e-05	100000	100	0.1000	0.0100	50	☑

Profile Likelihood (PL) and Hessian-based Confidence Intervals (CI)

	Parameter	Identifiability	Lower CI (PL)	Upper CI (PL)	Lower CI (Hessian)	Upper CI (Hessian)
1	S_to_pS	identifiable	1.2404	1.5282	1.2507	1.5102
2	dimerization	identifiable	0.1602	0.3663	0.1605	0.3252
3	nuc_import	identifiable	0.3721	1.7085	0.3760	1.0614
4	dimer_break		NaN	NaN	NaN	NaN
5	nuc_export	identifiable	0.4765	0.7929	0.4806	0.7307
6	S_C1		NaN	NaN	NaN	NaN
7	scale_pS_obs_C1		NaN	NaN	NaN	NaN
8	scale_S_obs_C1		NaN	NaN	NaN	NaN

Figure 1: **Profile Likelihood Estimation - User Interface.** All parameters for which a profile likelihood will be calculated are selected in the upper table. Boundaries for allowed parameter variations are specified, here four orders of magnitude around the current best fit value. Further settings like the maximum step number are left to their default value. The four analyzed parameters are all identifiable, as displayed in Fig. 2 and quantified in the lower table. Approximation of the confidence intervals using the Hessian-based Fisher-information matrix yields similar results as the numerical profile likelihood estimation, which is expected for identifiable parameters.

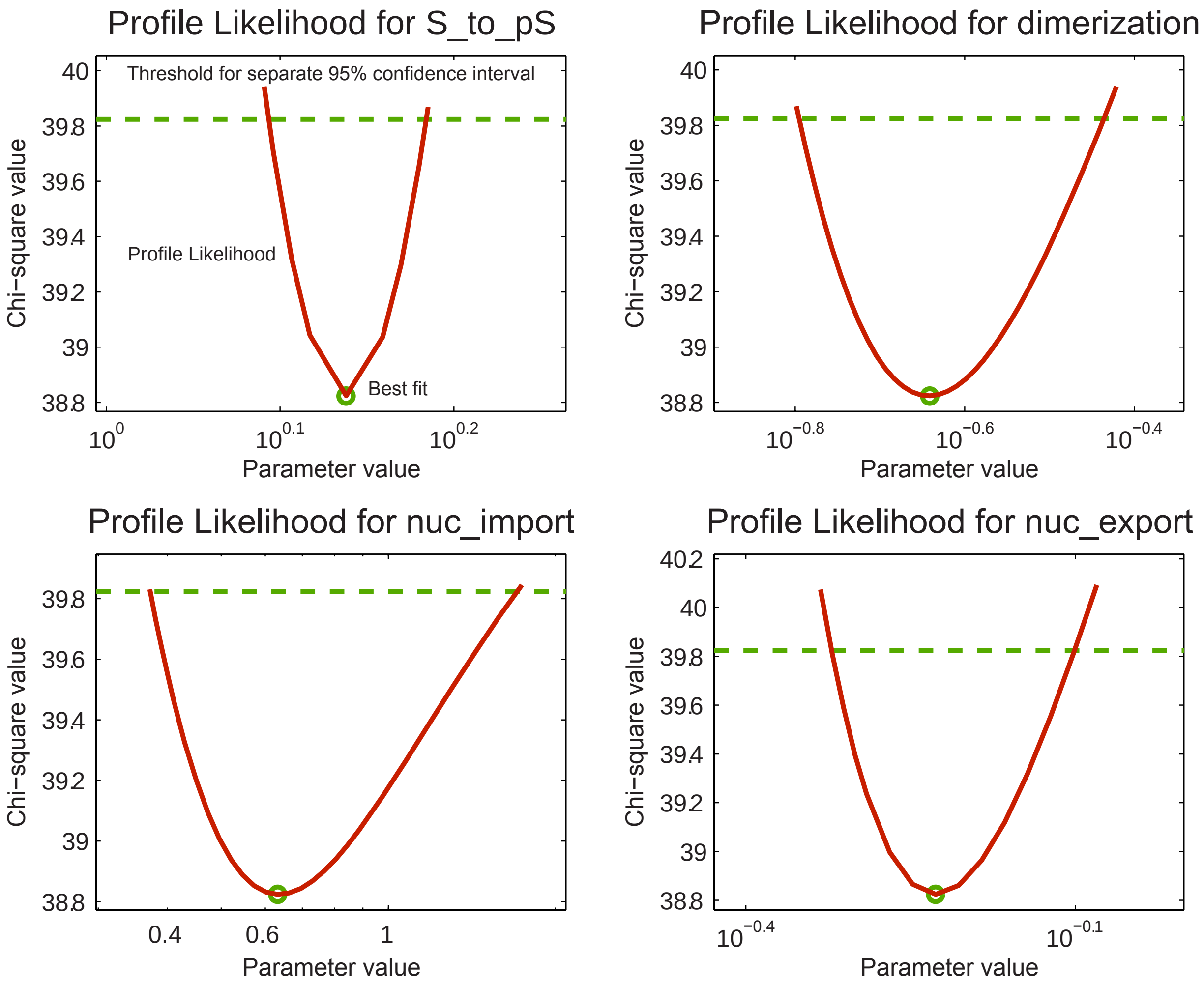

Figure 2: **Profile Likelihood Estimation - Results.** Starting from the best fit of the JAK-STAT model to dataset 1 of [1], depicted by the green circle in each subplot, the parameter of interested is systematically increased or decreased. After each step, all other parameters are calibrated by minimizing the least-square distance between model predictions and measured data. Since all four parameters are identifiable, the goodness-of-fit decreases resulting in an increased χ^2-value, i.e. increased profile likelihood (red solid line). Threshold crossing with a statistically predefined maximum chi-square value (green dashed line) corresponds to the upper or lower 95% confidence interval for the investigated parameter (compare [2]). All x-axes are in logarithmic scale.
